# YOLO-WAD for Small-Defect Detection Boost in Photovoltaic Modules

**DOI:** 10.3390/s25061755

**Published:** 2025-03-12

**Authors:** Yin Wang, Wang Yun, Gang Xie, Zhicheng Zhao

**Affiliations:** College of Electronic Information Engineering, Taiyuan University of Science and Technology, Taiyuan 030024, China

**Keywords:** photovoltaic module, YOLOv10n, defect detection, small-target inspection

## Abstract

The performance of photovoltaic modules determines the lifetime of solar cells; however, accurate detection remains a challenge when facing smaller defects. To address this problem, in this paper, we propose a YOLO-WAD model based on YOLOv10n. Firstly, we replace C2f (CSP bottleneck with two convolutions) with C2f-WTConv (CSP bottleneck with two convolutions–wavelet transform convolution) in the backbone network to enlarge the receptive field and better extract the features of small-target defects (hot spots). Secondly, an ASF structure is introduced in the neck, which effectively fuses the different levels of output features extracted by the backbone network and enhances the model’s ability to detect small objects. Subsequently, an additional detection layer is added to the neck, and C2f is replaced by C2f-EMA (CSP bottleneck with two convolutions–efficient multi-scale attention mechanism), which can redistribute feature weights and prioritize relevant features and spatial details across image channels to improve feature extraction. Finally, the DyHead (dynamic head) detection head is introduced, which enables comprehensive scale, spatial, and channel awareness. This greatly enhances the model’s ability to classify and localize small-target defects. The experimental results show that YOLO-WAD detects our dataset with an overall accuracy of 95.6%, with the small-target defect detection accuracy reaching 86.3%, which is 4.1% and 9.5% higher than YOLOv10n and current mainstream models, verifying the feasibility of our algorithm.

## 1. Introduction

Photovoltaic (PV) cells are a key technology for efficiently converting solar energy into electrical energy, thus becoming an integral part of the renewable energy sector [1]. However, in practice, PV panels may face a variety of potential failures and defects, such as hot spots, fractures, and vegetation shading. These problems not only lead to energy loss and reduce the power output of the cells [2,3] but may even cause system failures in extreme cases. Among them, hot-spot and crack defects are particularly important and considered to be one of the main causes of power loss in PV modules.

With the rapid development of computer vision and artificial intelligence technologies, defect detection methods based on these technologies have received increasing attention from researchers and practitioners. In particular, deep learning techniques have made significant breakthroughs in object detection and can learn higher-level feature representations [4], thus significantly improving the accuracy and robustness of defect detection.

Deep learning models can autonomously extract complex features and patterns from images by utilizing a wide range of datasets and neural network architectures, outperforming traditional methods; therefore, integrating computer vision and algorithms into defect detection systems offers a great deal of help in improving efficiency and reliability in industrial production [5].

Traditional object detection algorithms use low-level features (e.g., edge information) [6] to detect small objects, with poor performance. Therefore, the use of traditional object detection algorithms for defect detection in small objects is not known to be challenging.

In recent years, many scholars have made many efforts in small-target defect detection. In general, small-target defect detection methods can be categorized into the following three types: (1) using contextual information, (2) applying feature fusion, and (3) generating enhanced features. For example, Ref. [7] proposed a fully end-to-end object detector in which an object relationship module is designed to integrate contextual information of features. Ref. [8] proposed a trident network for detecting objects of different sizes, which utilizes Atros convolution with multiple dilation rates to generate different receptive fields in parallel. Ref. [9] proposed a feature pyramid network (FPN) that merges feature maps at different stages. To deal with the inconsistency between different feature scales, Ref. [10] proposed an adaptive spatial feature fusion method by learning weighting parameters. Ref. [11] proposed a path aggregation network by designing a bottom–up path augmentation branch, which fully integrates information from high-level features and low-level features.

Although existing methods have made significant progress in small-target defect detection, there are still some problems and challenges:

(1) Inconsistency between different feature scales is still a difficult problem, especially in multi-scale target detection, meaning that how to effectively fuse different levels of features still needs further research;
(2) Small targets occupy fewer pixels in an image, their feature expression ability is weak, and they are easily overwhelmed by background information, leading to a decrease in detection accuracy;
(3) In practical industrial applications, defect detection systems need to have high real-time performance, while some existing deep learning models still have room for improvement in terms of computational efficiency and real-time performance.

In order to further improve the accuracy and robustness of small-target defect detection, this paper proposes the YOLO-WAD algorithm based on YOLOv10n. YOLO-WAD derives its name from the letters in its key improvement modules: C2f-WTConv (W), ASF (A), and DyHead (D). The main contributions of this paper include the following:

(1) Instead of C2f, we use C2f-WTConv in the backbone part, and the main purpose of WTConv is to improve the accuracy of small-target detection for PV modules by processing different frequency bands of the input data, using the wavelet transform, expanding the convolutional sensory field through a multi-frequency response, and performing small-kernel convolution operations in different frequency ranges.
(2) We apply an attention scale sequence fusion (ASF) structure to the neck layer, which further strengthens the ability of the model to recognize the details of small objects. By merging spatial and multi-scale features, the ASF structure efficiently fuses the output characteristics of different layers (P2, P3, P4, and P5) extracted from the backbone network. This efficient feature fusion strategy enhances the model’s ability to detect small objects and improves the overall feature representation of the model.
(3) In the detection head part, we introduce the dynamic head framework DyHead, which combines the object detection head with the attention mechanism to enhance the model’s ability to classify and localize objects and better detect the absence of small targets, thus improving the detection accuracy.
(4) We integrate the C2f-EMA structure into the network using an efficient multi-scale attention module embedded into C2f. This enhancement improves feature extraction by redistributing feature weights, prioritizing relevant features and spatial details across image channels. As a result, it enhances the network’s ability to detect targets of different sizes.

## 2. YOLO-WAD Algorithm

### 2.1. YOLO-WAD Structure

**Figure 1:**
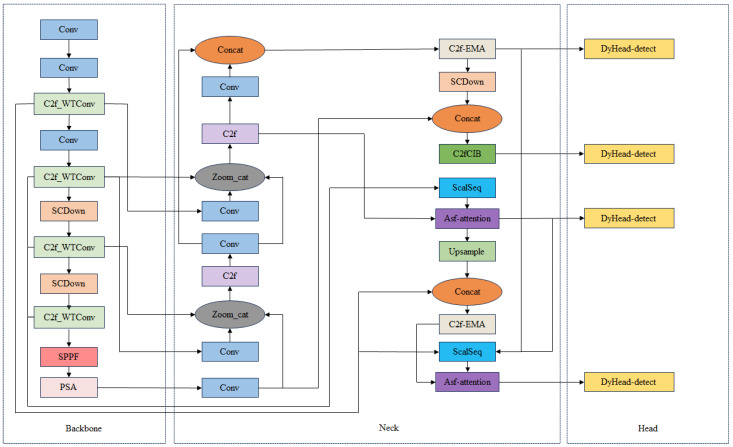
YOLO-WAD algorithm structure.

Aiming to achieve the difficult detection of small-target defects in PV modules, based on YOLOv10n, we propose an improved YOLO-WAD algorithm. The specific network structure is shown in Figure 1.

### 2.2. Wavelet Transform Convolution (WTConv)

The WTConv module [12] utilizes the wavelet transform (WT) and inverse wavelet transform (IWT) to process the multi-scale information of the input image, thereby enhancing the model’s capability in detailed feature extraction, especially for the detection of small defects on the surface of PV modules. In this study, we integrate WTConv into the backbone system of the baseline detector to extract the small-target defect information to further improve the performance of PV module defect detection.

The Haar wavelet transform decomposes an image into low- and high-frequency components in each spatial dimension by deep convolution and downsampling. While the one-dimensional Haar WT can be represented by the convolution kernels [1, 1]/√2 and [1, −1]/√2, the two-dimensional Haar WT extends these operations to two dimensions, resulting in four filters:

(1) $$f_{LL}=\frac{1}{2}\left[\begin{array}{cc} 1 & 1\\ 1 & 1 \end{array}\right],f_{LH}=\frac{1}{2}\left[\begin{array}{cc} 1 & -1\\ 1 & -1 \end{array}\right],f_{HL}=\frac{1}{2}\left[\begin{array}{cc} 1 & 1\\ -1 & -1 \end{array}\right],f_{HH}=\frac{1}{2}\left[\begin{array}{cc} 1 & -1\\ -1 & 1 \end{array}\right]$$

The f stands for filter, *L* for low frequency, and *H* for high frequency, while $f_{LL}$, $f_{LH}$, $f_{HL}$, $and\;f_{HH}$ represent low-frequency–low-frequency filters, low-frequency–high-frequency filters, high-frequency–low-frequency filters, and high-frequency–high-frequency filters, respectively.

These filters further decompose the input image X into a low-frequency component $X_{LL}$ and high-frequency components $X_{LH}$, $X_{HL}$, and $X_{HH}$.

**Figure 2:**
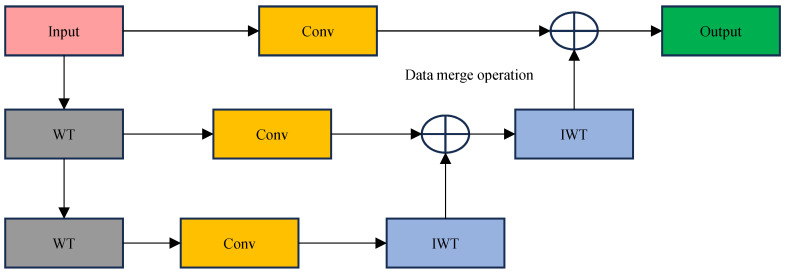
An example of the WTConv operation on a single channel using two-level wavelet decomposition.

Figure 2 illustrates the WTConv module with a two-stage wavelet transform. The process can be summarized as follows:

Firstly, the input image is first processed into different frequency components, which are decomposed into low- and high-frequency features using the wavelet transform. The low-frequency features retain the general structural information of the image, while the high-frequency features capture the details and edge information in the image. Next, the decomposed features are subjected to a convolution operation to further extract useful information.

The result of each layer of wavelet decomposition is reconstructed by inverse wavelet transform after the convolution process to recombine the feature information of each layer. The final output is the feature map after multi-layer decomposition, convolution, and fusion, which retains the global information of the image and highlights the detailed parts, especially for the features of small-target defects of PV modules. The specific calculation is shown in Equation (2):

(2) $$\gamma =IWT(Conv\left(w,WT\left(X\right))\right)$$

where γ represents the feature map of the final output, X denotes the input tensor, *w* denotes the convolution kernel weights, and Conv, WT, and IWT denote the convolution operation, wavelet transform, and its inverse transform, respectively.

**Figure 3:**
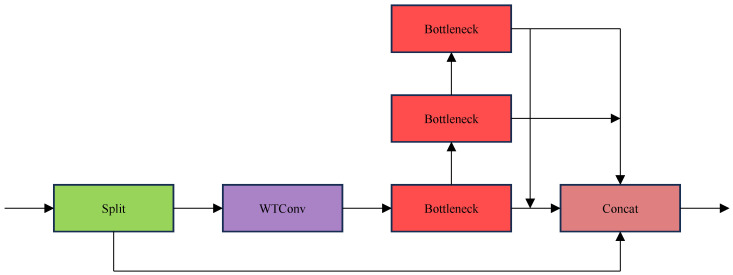
C2f-WTConv.

In this paper, we introduce C2f-WTConv as an alternative to C2f, as shown in Figure 3. C2f-WTConv includes initial input decomposition, WTConv, bottleneck, and Concat modules, which are coordinated to ensure the sensitivity of the detection task to small-target defects, which is very suitable for PV module defect detection scenarios.

Both the C2f module and the WTConv module demonstrate their unique value in PV module defect detection, as the C2f module is robust in its ability to maintain stable performance in the face of our PV module defect detection dataset, regardless of changes in the data acquisition environment, acquisition equipment, and other factors, and does not fluctuate significantly due to data variations. As for WTConv module, it is good at handling data with specific frequency characteristics. Different types of defects in photovoltaic module image data often show unique characteristics at specific frequencies, and the WTConv module is good at capturing this key information, laying the foundation for accurate defect identification.

When we combine C2f and WTConv to form the C2f-WTConv module, the advantages of the C2f-WTConv module are fully upgraded. In the real-world scenario of PV module defect detection, it is perfectly adapted to PV module image data from different sources and with different characteristics. Whether it is images collected under different lighting conditions or images of PV modules from different production batches, it can accurately analyze them to determine whether there are defects or not, which greatly reduces dependence on specific data distribution, thus making the whole inspection model more stable and reliable.

In terms of its ability to capture image features, the C2f module, through its carefully designed structure, is able to keenly detect subtle local features in PV module images, so that even very small defect details cannot escape its ‘eyes’. The WTConv module, because of its enlarged sensing field and its ability to process different frequency bands, not only focuses on small-target defects but also grasps the overall structural information of the PV module from a global perspective, identifying fine structures hidden in global features, such as subtle anomalies between defects and the background in the detection of defects in the PV module. The C2f-WTConv module combines the strengths of the two and is able to capture both C2f-WTConv modules, combining the strengths of both and achieving a perfect balance between capturing fine local defect features and good information about the global module structure. This is extremely beneficial for the detection of small-target defects in PV modules, as well as for improving the overall inspection accuracy, allowing us to more accurately detect small defects in PV modules which are easily overlooked and safeguarding the quality and performance of PV modules.

### 2.3. Attentional Scale Sequence Fusion (ASF)

In this paper, the ASF architecture [13] is introduced into the neck layer in order to better capture information about small targets of photovoltaic modules. ASF is a framework for combining spatial and multi-scale features in object detection and segmentation, allowing the fusion of output features extracted from the P2, P3, P4, and P5 layers of the backbone network. This feature fusion network architecture consists of two components that can provide complementary information for target defect object segmentation: the SSFF module combines global information from multi-scale images, while the TFE (temporal feature extraction) module captures local fine details of small target objects. So the combination of the two allows for the better detection of small-target defects both locally and globally.

**Figure 4:**
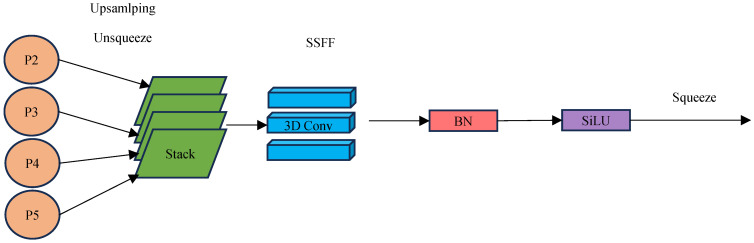
Structure of the SSFF module.

The SSFF (spatial-scale feature fusion) module is designed to integrate multi-scale spatial features into a unified representation, as shown in Figure 4. It handles features at multiple levels (e.g., P2 to P5) by first upsampling or downsampling features to a consistent scale. These multi-scale features are then stacked together for deeper integration. Apply 3D convolutional layers to capture the interdependencies between spatial dimensions and feature levels. Batch normalization (BN) ensures stable training, while the SiLU (sigmoid-weighted linear unit) activation function enhances the nonlinearity of the network. The module outputs fused features that combine information from multiple spatial scales to improve the representation of downstream tasks. The scaled image of the input SSFF can be obtained from Equations (3) and (4):

(3) $$F_{\sigma }\left(w,\;h\right)=G_{\sigma }(w,\;h)\times f(w,\;h)$$

(4) $$G_{\sigma }\left(w,\;h\right)=\frac{1}{2\pi \sigma^{2}}e^{-(w^{2}+h^{2})/2\sigma^{2}}$$

where f(w,h) denotes a 2D input image of width w and height h. $F_{\sigma }(w,\;h)$ is generated by smoothing through a series of convolutions with a 2D Gaussian filter $G_{\sigma }(w,\;h)$. Here, σ represents the scale parameter that determines the standard deviation of the 2D Gaussian filter used in the convolution process.

**Figure 5:**
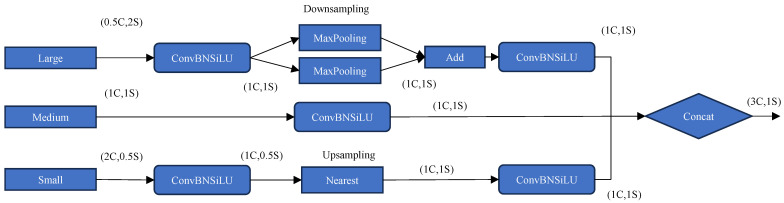
TFE module structure.

The TFE module focuses on fusing temporal features from large, medium, and small receptive fields, as shown in Figure 5. It uses three branches to process inputs at different scales. The large branch uses a combination of convolution, batch normalization, SiLU activation, and maximum pooling, followed by an additive operation to preserve key features. The medium branch uses the convolutional layer directly to process the input. The small branch involves upsampling, followed by a convolutional layer to optimize details. Finally, all processed outputs are concatenated to create a comprehensive representation of temporal features that effectively captures global and local temporal dependencies. As shown in Equation (5), three feature maps of the same size—large, medium, and small—are convolved once and then spliced in the channel dimension.

(5) $$F_{TFE}=Concat(F_{l},F_{m},F_{s})$$

where $F_{TFE}$ denotes the feature map output from the TFE module, and $F_{l}$, $F_{m},$ and $F_{s}$ correspond to large, medium, and small feature maps, respectively.

The ASF module shows significant innovations in PV module defect detection. First, the introduction of the attention mechanism can accurately locate small-target defect areas, strengthen the extraction of their features, and overcome the problem of background interference in traditional methods. Secondly, in the scale sequence fusion, the image is first decomposed at multiple scales and then fused with different scale features, so that the details are grasped at a small scale, and the overall relationship is grasped at a large scale, thus solving the limitation of single-scale detection. Third, its sequence fusion is unique, simulating the observation process of human vision from the whole to the local, locating the general area of defects first and then focusing on the details to improve the accuracy and efficiency of detection.

### 2.4. Dynamic Detection Head

In the YOLOv10 model, the detection head is unable to effectively detect and localize small objects in the PV assembly due to the large receptive field of the network. DyHead [14] is a dynamic head framework designed for object detection. It introduces an attention mechanism across feature layers, spatial locations, and channels to achieve comprehensive scale, spatial, and task awareness, which significantly improves the performance of small object detection.

**Figure 6:**
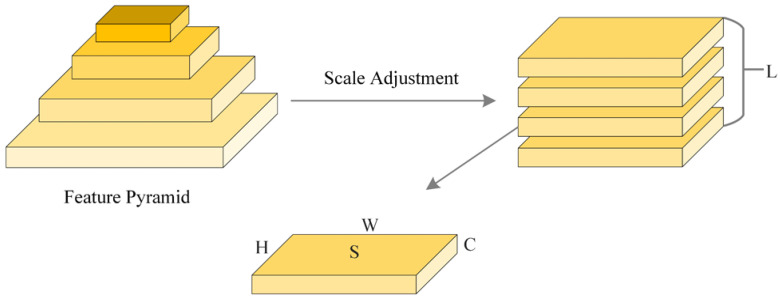
An illustration of the DyHead approach.

Figure 6 illustrates the core structure of the DyHead method and its role in feature processing: (1) The pyramid shape on the left side represents feature maps from different layers, with the bottom layer’s features being larger and containing more spatial information, and the top layer’s features being smaller and emphasizing higher-level semantic information. This design is similar to looking at an object in a way that focuses on the whole (low-level features) while capturing local details (high-level features). (2) Regarding scale adjustment, the arrows in the figure indicate that, after extracting features from the layers of the pyramid, they are transformed into feature maps with the same dimensions through a uniform scale adjustment operation. This process allows features of different scales to be fused and processed equally in subsequent steps without the loss of information or mismatch due to size differences. (3) For multi-layer processing (L feature layer stacking), the adjusted feature maps are stacked into multiple layers (indicated by the parallel rectangles on the right) to form a combination of features that can capture spatial, channel, and scale information simultaneously. This structure ensures that the model can handle targets of various sizes and shapes in complex scenes, where both large background information and small detailed features are attended to. (4) Regarding the basic feature structure (H, W, and C), the bottom of the figure shows the basic structure of the feature map: H for height, W for width, C for the number of channels, and S for spatial scale information. By adjusting the scale, the model can flexibly adapt to different sizes of inputs while retaining key information.

**Figure 7:**
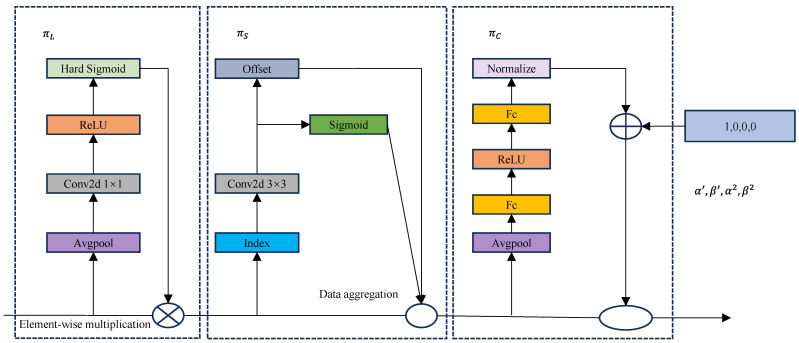
DyHead block structure.

Figure 7 shows the structure of the DyHead module.

The $\pi_{L}$ module focuses on feature weight assignment in the channel dimension. The input features are extracted from the global information by average pooling, and then, the weights are generated by convolution (Conv2d 1 × 1), ReLU, and hard sigmoid functions. Ultimately, these weights act on the channel dimensions of the feature map, dynamically adjusting the response strength of different channels to highlight more useful information.

The $\pi_{S}$ module mainly deals with the spatial dimension of the feature map. It first senses the spatial details of the target through an offset mechanism and subsequently generates spatial weights using sigmoid functions. These weights are dynamically adjusted to the input feature maps to enhance the salient regions of the spatial dimension, which is particularly suitable for capturing small defects on PV modules.

The $\pi_{C}$ module combines global context and local feature information. The input features are first passed through the fully connected layer (Fc), activation function (ReLU), and normalization to generate the integrated features. Meanwhile, the feature magnitude is adjusted by an exponential function to enhance the contrast between the local information and the global context, and improve the recognition of small targets.

$\pi_{L}$ stands for the scale-aware attention mechanism, which can dynamically fuse features of different scales according to their semantic importance. $\pi_{S}$ stands for the spatial-aware attention mechanism, which can continuously pay attention to spatial locations and discriminative regions co-existing in the feature layer. $\pi_{C}$ stands for the channel-aware attention mechanism, which is able to co-learn and generalize different representations for different objects.

Dynamic detection head module innovation

Regarding dynamic adaptation, according to the input image characteristics, it can flexibly adjust its own structural parameters in real time, such as dynamically changing the size of the convolution kernel and the number of layers in order to meet the needs of the detection of defects at different scales of small targets, breaking through the limitations of the traditional fixed structure of the detection head.

For multi-feature fusion, it can intelligently fuse the features extracted from different layers and different sensory fields to comprehensively capture the details and contextual information of small-target defects and enhance the ability to discriminate small targets in complex backgrounds.

Regarding efficient learning, the feature-learning mechanism in the training process is optimized to quickly focus on the key features of small targets and reduce redundant learning, which improves the detection speed and accuracy at the same time, making the detection of small-target defects in PV modules more efficient and reliable.

### 2.5. Embedding Efficient Multi-Scale Attention Mechanisms in C2f

The C2f module in YOLOv10 dynamically adjusts the number of channels through splitting and concatenation operations to optimize feature extraction while controlling computational complexity, thus improving gradient flow detection accuracy [15]. It combines convolutional and residual structures to reduce network training and solve the gradient vanishing problem for improved feature extraction.

**Figure 8:**
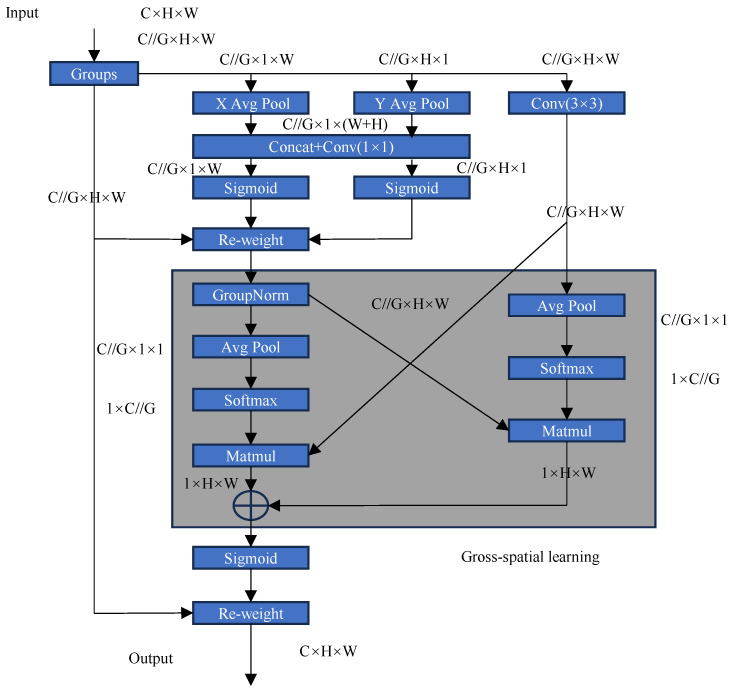
Efficient multi-scale attention mechanism.

The C2f-EMA module presented in this paper enhances feature extraction by redistributing feature weights using the EMA [16] attention mechanism. Figure 8 illustrates the workflow of an efficient multi-scale attention mechanism whose main objective is to better capture features of targets at different scales in an image, especially in the detection of small targets in PV modules, where this mechanism can provide significant advantages.

Firstly, the input features are divided into different groups for parallel processing, and each group is subjected to a convolution operation to extract a preliminary feature representation. The features are then further fused by a core module (e.g., concatenated Conv) to generate more representative multi-scale information.

Next, the features are fed into an attention branch that contains several important operations: average pooling and global pooling are able to summarize the significance of the features from different perspectives, while matrix operations (e.g., the cross-connection parts of the figure) combine the information from the spatial dimension with the information from the channel dimension. The results of these operations are adjusted by weighting (Re-weight) and the activation function (sigmoid) and used to enhance the key features of the small targets.

**Figure 9:**
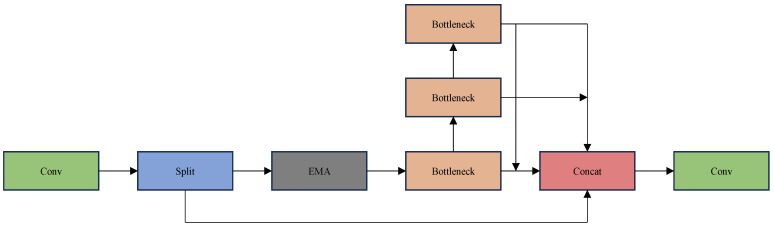
C2f-EMA.

In this paper, we introduce C2f-EMA as an alternative to C2f that utilizes the EMA structure to redistribute the feature map to assign higher weights to more relevant features and spatial details in the image. Figure 9 illustrates the structure of the C2f-EMA module, which optimizes the performance of small-target defect detection for PV modules through clever feature segmentation, extraction, and fusion strategies: 1. The first step is splitting and concatenating parts that take into account global and local information. Indeed, the C2f-EMA module retains the global information of the input by splitting the path when extracting the features and, at the same time, reinforces the detailed representation of the small targets through the bottleneck module. This allows the module to both detect large-scale defects on the surface of PV modules and accurately capture small-target defects. 2. The bottleneck part is more adaptable to small-target detection. Indeed, the bottleneck module and the branching structure are extremely capable of capturing multi-scale features, which can cope well with the problem of changing target sizes in the detection of small targets of PV modules. 3. The computational efficiency is high, making it suitable for large-scale detection tasks: by splitting the path to reduce redundant calculations, combined with the lightweight bottleneck design, the module improves the detection accuracy while significantly reducing the computational cost, which is very suitable for batch photovoltaic module detection in industrial sites. 4. The module is characterized by strong feature expression and better anti-interference ability. Indeed, after fusing global and local features, the module can effectively deal with the interference of the complex background on the surface of the PV module, which makes it easier to identify the defects of small targets. Overall, the C2f-EMA module provides strong technical support for the detection of small defects in PV modules through its clever and efficient structural design, which not only improves the accuracy of the detection but also ensures efficiency and robustness in industrial applications.

C2f-EMA is innovative in several ways, outlined below.

The first is related to structural innovation, as the C2f module fuses multi-scale features and EMA optimizes model parameter updates. The combination of the two allows the model to learn small-target defect features more stably without adding too much computation, achieving a balance between a light weight and efficiency.

Then, to improve detection capability, C2f initially extracts multi-scale features of small targets, and EMA adjusts and optimizes the model to strengthen the capture of subtle defect features, effectively avoiding the model from falling into local optimality and significantly improving the detection accuracy.

The third innovation is related to robustness and generalization enhancement. In the face of complex environments, such as uneven lighting and angle changes, the module is highly adaptable. It also maintains good detection performance on datasets with different defect types by virtue of its unique structure and EMA mechanism.

There, ‘//’ is the integer division operator, and G is the grouping number. C (Channels): indicates the number of channels. H (Height): represents the height. W (Width): indicates the width. 

## 3. Results

### 3.1. Experimental Environment

In order to complete the experiment, we carried out a study based on the PyTorch framework. The experimental environment was configured as follows: the operating system was Windows, the Python version was 3.8, the PyTorch version was 1.11.0, and the experimental hardware was NVIDIA RTX 3080 Ti.

In this experiment, the stochastic gradient descent (SGD) optimizer was used for model optimization and training. For the SEM-YOLO network parameters, the initial settings were set to an input image size of 640 × 640 and an initial learning rate of 0.01, and the learning rate was dynamically adjusted using the OneCycleLR strategy to ensure the stability of the training process. In order to smoothly guide the model to learn and obtain a better initialization state at the beginning of training, we designed a warm-up strategy with a duration of three epochs. The batch size of the experiment was set to 16, and the total training period was planned to be 300 epochs.

When training neural networks, gradient descent algorithms are commonly used to optimize the model parameters, and the learning rate is one of the key hyperparameters, controlling the step size of the parameter update. By calculating the gradient of the loss function over the parameters, we can specify the direction of parameter adjustment, and the learning rate determines the magnitude of each adjustment along a given direction. If the learning rate is too large, the model will easily skip the optimal solution, resulting in unstable training, non-convergence, and large fluctuations or even an increase in the loss function; if the learning rate is too small, the model will converge slowly and take a lot of time to train. For example, for a simple linear regression model, if the learning rate is set to 0.1, the pace of parameter update is large; if the learning rate is set to 0.001, the pace is small, and the training process is slowed down. We set the learning rate to take the middle value, 0.01, so the update would neither be too fast nor too slow.

When training the model, we did not use the whole dataset to calculate the gradient and update the parameters but split it into small batches, and the number of samples used in each iteration was the batch size. The batch size was large, so it could calculate the gradient by integrating information from more samples, leading to more accurate results, more stable training, and a better convergence effect of the model. However, this occupied more memory, each round of training took a long time, and the generalization ability of the model was worse. For example, if the batch size is set to 16, each iteration will take 16 samples from the dataset to calculate and update the parameters.

### 3.2. Datasets

**Figure 10:**
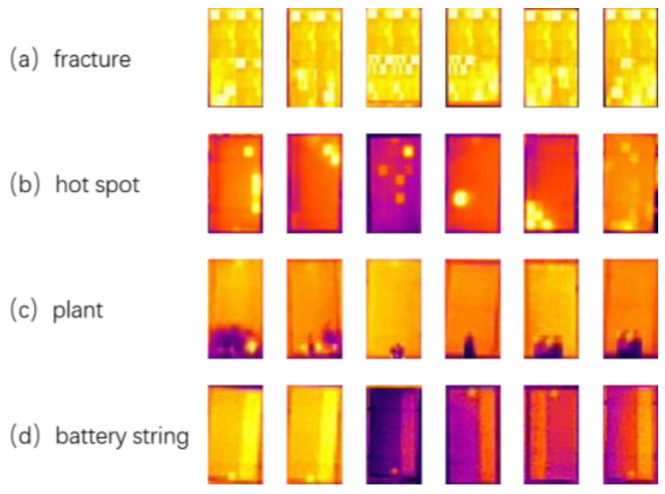
Defect categories.

During the experiment, the dataset was sourced from a collaborative enterprise. They deployed a hexacopter UAV outfitted with a thermal infrared lens along the site inspection route. A total of 2272 infrared aerial images were captured at a low altitude (ranging from 10 to 15 m above the PV module) in a centralized manner. These images were then carefully screened and served as the raw data for the experiment, 2037 of which were used for training, 224 for validation, and 11 as the test set. For these images, we manually annotated them using the LabelImg tool, and a total of four defect types were noted, including fracture, hot spot, plant, and battery string, as shown in Figure 10.

**Figure 11:**
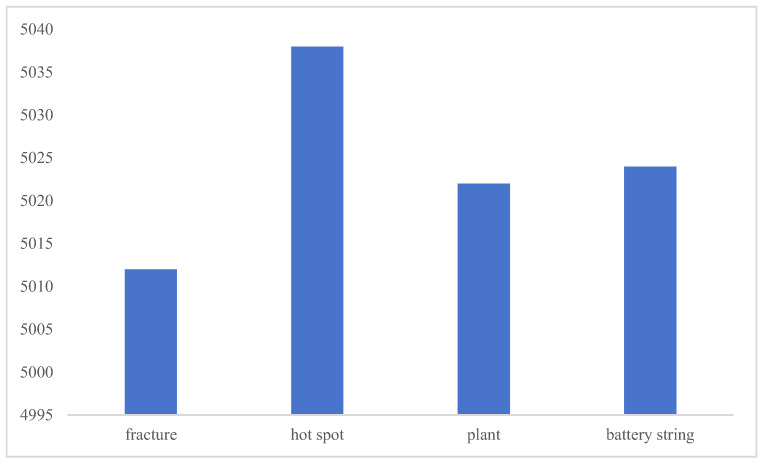
Label distribution.

To address the sample imbalance, we set a target number of 5000, undersampling ‘hot spots’ by a large amount, from 36,524 to about 5000; undersampling ‘plants’ by a small amount, from 9567 to about 5000; oversampling ‘battery strings’ by a small amount, from 5327 to about 5000; and oversampling ‘fracture’ by a large amount, from 835 to about 5000. All annotations were stored in YOLO format, as shown in Figure 11.

### 3.3. Evaluation Indicators

In our experiments, we used the metrics of detection precision mAP (mean average precision), accuracy precision, and recall to evaluate the model performance [17].

Precision: The ratio of the number of correctly retrieved samples to the total number of retrieved samples was used to measure the precision of the model.

Recall: The ratio of the number of correctly retrieved samples to the total number of samples that should have actually been retrieved was used to reflect the coverage ability of the model.

AP (average precision): The area under the PR curve (recall on the horizontal axis and precision on the vertical axis) of a category was used to measure the single-category detection performance.

MAP (mean average precision): The average of all categories of AP was an important indicator for evaluating the overall detection performance of the model.

The calculation expression of each evaluation index is as follows:

(6) $$P=\frac{TP}{TP+FP}$$

(7) $$R=\frac{TP}{TP+FN}$$

(8) $$AP=\int_{0}^{1}precision\left(recall\right)d\left(recall\right)$$

(9) $$mAP=\frac{1}{N}\sum_{i=1}^{N}{AP}_{i}$$

True positive (TP) indicated the number of targets detected correctly, i.e., the number of instances that the model correctly recognized as targets.

False positive (FP) indicated the number of targets detected incorrectly, i.e., the number of instances where the model incorrectly identified a non-target as a target.

False negative (FN) indicated the number of targets that were missed, i.e., the number of instances where the model failed to recognize a target.

N indicated the total number of detected object categories, used to calculate the mean value of mAP.

### 3.4. Experimental Results and Analysis

#### 3.4.1. Comparison with Other Algorithms

In this study, YOLOv10n was used as the basis for improvement, and in order to verify the effectiveness of the proposed YOLO-WAD algorithm, we selected a variety of existing mainstream models for comparison.

We compared the YOLO-WAD model with YOLOv8n, YOLOv9s, YOLOv10n, YOLOv11, and RT-DETR series models. The following reasons were those mainly taken into account during the selection process.

YOLOv8n, YOLOv9s, YOLOv10n, and YOLOv11, which are models belonging to the same YOLO series, were selected for comparison for the following main reasons: Firstly, they are extremely well known and widely used in the field of target detection, and they have excellent performance in many scenarios of small-target detection. For the task of detecting small-target defects (hot spots) in PV modules, our YOLO-WAD model is based on the YOLO architecture with targeted improvements. A comparison of the models in the same series clearly showed the innovations and optimizations we made to address the specific needs of PV module inspection scenarios, such as complex lighting conditions, special textures and structures of the modules, etc. For example, the improvements in the feature extraction module for small targets enabled our model to more accurately capture the unique features of small targets such as hot spots, which had not been considered by the other models in the same series.

The RT-DETR family of models was chosen for comparison because of its unique technology path. It is based on the transformer architecture for end-to-end detection, which is fundamentally different from the traditional YOLO series based on convolutional neural networks. In real-world PV module detection environments, where the background is highly complex and small-target hot spots may be obscured by the surrounding normal module areas or other interfering factors, RT-DETR series models have an advantage in dealing with such complex scenarios, as they are able to analyze the entire PV module image more comprehensively thanks to the transformer’s powerful global modeling capabilities. By comparing with the RT-DETR series models, we explored the performance differences between the different technology routes in the detection of small-target defects in PV modules, such as the detection accuracy, recall rate, and the accuracy of locating small targets in complex backgrounds, so as to evaluate the performance and application potential of the YOLO-WAD model in a more comprehensive way.

The existing mainstream models RT-DETR-l, RT-DETR-x, RT-DETR-Resnet50, RT-DETR-Resnet101 [18], YOLOv9s [19], YOLOv8n [20], and YOLOv11 [21] were compared. The MAPs detected in our dataset were 81.4%, 81.4%, 86%, 87.5%, 92.7%, 91.57%, and 91.6%, respectively, as shown in Table 1, and 92.7%, 91.57%, and 91.6%. Among them, the small-target defect detection accuracies were 64.6%, 61.9%, 68.5%, 71%, 78.1%, 75.38%, and 75.2%, respectively. It can be seen that these models performed poorly in detecting small-target defects in PV modules, and the overall accuracy of the detection of small-target defects was low. Meanwhile, our model, YOLO-WAD, achieved an accuracy of 86.3% for the detection of small targets and was ahead of current mainstream models.

Table 1 shows a clear comparison of the algorithms, leading to the below conclusions.

YOLO Series:

YOLOv8n had the best performance in precision (91.38%) and mAP@0.5 (91.57%) but was slightly below YOLOv9s in recall (84.96%).

YOLOv9s had the best performance in recall (89.44%) and mAP@0.5 (92.7%) but was slightly below YOLOv8n in precision (85.38%).

YOLOv10n showed balanced performance in precision (90.7%) and recall (88.14%), but the mAP@0.5 (91.5%) slightly lower than in YOLOv9s.

YOLOv11 showed stable performance in precision (89.89%) and recall (88.41%), but the mAP@0.5 (91.6%) was close to YOLOv10n.

RT-DETR Series:

RT-DETR-l and RT-DETR-x showed poor performance in precision, recall, and mAP@0.5, especially in hot-spot detection (64.6% and 61.9%).

RT-DETR-Resnet50 and RT-DETR-Resnet101 showed improved performance but still lower than the YOLO series in hot-spot detection.

We analyzed the reasons for the high and low accuracy of these algorithms.

YOLO series algorithms generally had higher accuracy in the fracture and battery string categories, possibly due to the features of these two types of defects being relatively stable and apparent and the feature extraction and detection mechanism of YOLO series algorithms being able to capture these features better. For example, the linear texture of a fracture and the regularly arranged structure of a battery string are well suited to the YOLO algorithm’s multi-layer feature fusion and fast detection strategy.

RT-DETR-Resnet series algorithms had a higher accuracy in the fracture category. ResNet’s residual structure helped extract deep image features, and a fracture with apparent structural changes could be better localized and identified by learning the residual information.

YOLO-WAD (our algorithm), overall, was leading in all indicators. It is speculated that it adopts an innovative network architecture design, which may incorporate a more effective attention mechanism to make the model focus on small-target defect features and, in data processing, improve the model’s ability to detect small targets in actual scenes with complex lighting and occlusion through unique data enhancement and preprocessing strategies.

For RT-DETR-l and RT-DETR-x, the overall precision and recall were low. On the one hand, this might have been because the number of parameters and complexity of the model had not been set reasonably, and the feature extraction was not sufficient when dealing with small-target defects (e.g., hot spot), leading to more missed and false detections. On the other hand, the lack of effective contextual information utilization mechanisms made it difficult to distinguish between target and background, reducing the detection accuracy.

For YOLOv9s, the accuracy rate was lower compared to other YOLO versions. The hyperparameter adjustment might not have been optimal during the network optimization process, which made the model’s ability to distinguish between positive and negative samples in the detection process decrease, thus affecting the accuracy rate.

The YOLO series of algorithms performed well in the PV module defect detection task, especially our YOLO-WAD algorithm, which had a significant advantage in small-target defect detection. By optimizing the model architecture and training strategy, YOLO-WAD effectively improved the detection accuracy and robustness, providing reliable technical support for the quality inspection of PV modules. Future research can further explore how to reduce the computational resource requirements to adapt the algorithm to a wider range of application scenarios.

#### 3.4.2. Visualization and Analysis

**Figure 12:**
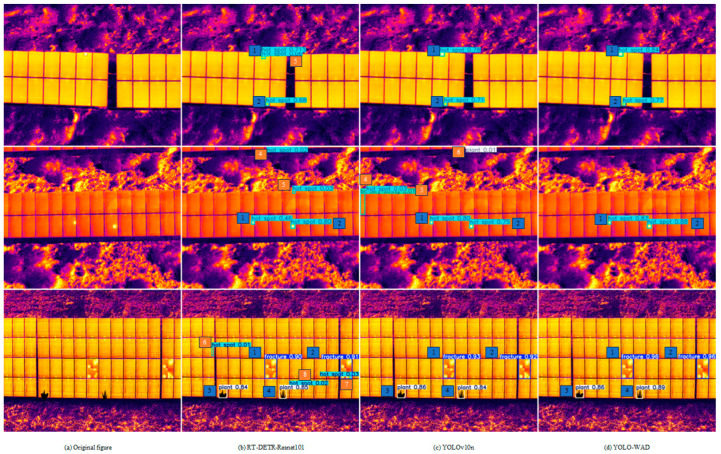
Detection performance of different algorithms on the same detected image.

To visually demonstrate the superiority of the algorithms in this paper, Figure 12 compares the detection results of the RT-DETR-Resnet101, YOLOv10n, and YOLO-WAD algorithms on the test set. The blue numbers in the graph indicate normal detection results, and the orange color represents a false detection situation.

Figure 12a shows the original image, which contains two hot-spot defects at the top of the original image. From the enlarged image, it can be seen that the detection results of RT-DETR-Resnet101 in Figure 12b are 0.713 and 0.68, as shown by numbers 1 and 2 in the figure, while number 5 is a misdetection, misdetecting the background color of the photovoltaic panel as a hot spot. The detection results of YOLOv10n in Figure 12c are 0.78 and 0.71, as shown by numbers 1 and 2 in the figure. The detection results of YOLO-WAD in Figure 12d are 0.84 and 0.77, as shown by numbers 1 and 2 in the figure. It is clear that the YOLO-WAD algorithm is more effective than the other algorithms in detecting the small-target defects with a high accuracy.

The original image in Figure 12a also contains two defects in the middle. The detection results of RT-DETR-Resnet101 in Figure 12b are shown as numbers 1 and 2 in the figure, which are 0.46 and 0.80, respectively, from which it can be intuitively seen that there are two misdetections, indicated by numbers 4 and 5 in the figure, whereby number 5 is a misdetection of one of the PV panel backgrounds as a hot spot, and number 4 is a misdetection of one of the images in the background as a hot spot. The detection results of YOLOv10n in Figure 12c are 0.80 and 0.75, as indicated by numbers 1 and 2, but there are three misdetections, as indicated by numbers 4, 5, and 6 in Figure 12c, where number 4 is the misdetection of one of the PV modules as a hot spot, number 5 is the misdetection of one of the PV modules as a battery string, and number 6 is the misdetection of one of the images in the background as a plant. The detection results of YOLO-WAD in Figure 12d are shown as numbers 1 and 2 in the figure, which are 0.83 and 0.88, respectively. These figures show that YOLO-WAD has higher confidence and stability in detecting defects in small targets, with no false detection.

In addition, the original image at the bottom of Figure 12a shows four defects, including two plants and two fractures. In Figure 12b, the detection results of RT-DETR-Resnet101 for the two plants are 0.84 and 0.85, as shown by numbers 3 and 4 in the figure, and the detection results for the fractures are 0.90 and 0.91, as shown by numbers 1 and 2 in the figure, while three false detections occur, which are the corresponding parts in Figure 6, Figure 7 and Figure 8, and all three false detections were due to the PV module image being mistakenly detected as a hot spot. In Figure 12c, the detection results of YOLOv10n for the two plants are 0.86 and 0.84, as shown by numbers 3 and 4 in the figure, and the detection results of YOLOv10n for the two fractures are 0.93 and 0.92, as shown by numbers 1 and 2 in the figure. In Figure 12d, the detection results of YOLO-WAD for the two plants are 0.86 and 0. 89, as shown by numbers 3 and 4 in the figure, and the detection results of YOLOv10n for the two fractures are 0.96 and 0.96, respectively, as shown by numbers 1 and 2 in Figure 12d; these data show that YOLO-WAD not only exhibits high confidence and stability in detecting small-target defects but also high accuracy in detecting other defects (e.g., plants and fractures), reflecting our algorithm’s superiority in overall and small-target defect detection.

RT-DETR-Resnet101 detected defects, showing a total of six misdetections, all of which were caused by the detection of small-target defects. We analyzed the general reasons for this, which are as follows: (1) Since the background of the object to be detected is often complex, small-target defects are easy to confuse with similar elements in the background. (2) The receptive field settings of RT-DETR-Resnet101 may not be fully adapted to small-target defects. (3) The sensory field settings of RT-DETR-Resnet101 may not be fully adapted to small-target defects. Small targets occupy few pixels in an image, and when the receptive field of a network is too large, it contains too much background information, resulting in the dilution of small-target features, making it difficult to focus on key ones. However, if the receptive field is too small, it is impossible to obtain the complete contextual information of small targets, which increases the possibility of misdetection. (4) The way in which the fusion of features at different levels in RT-DETR-Resnet101 takes place and its effects affect the detection of small-target defects. There were three misdetections in YOLOv10n’s defect detection performance, due to the following two aspects: (1) Although YOLOV10n has multiple detection heads, they may still be insufficient for dealing with small-target defects. For example, after multi-layer downsampling, the features of small targets may become too abstract and fuzzy, losing some key location and detail information. (2) In addition, the small-target defects themselves are of low resolution, occupying few pixels in the image and containing limited information such as texture and shape. This makes it difficult for the neural network to extract enough effective features to accurately describe the small-target defects, which leads to false detection.

The YOLO-WAD algorithm proposed in this paper showed significant advantages in practical applications. It not only performed well in small-target defect detection accuracy but also achieved higher performance in the overall defect detection accuracy index, while avoiding the phenomenon of false detection. This shows that the algorithm can not only meet the actual needs of industrial production but also provide new ideas and research directions for the future of small-target defect detection in PV modules.

#### 3.4.3. Ablation Experiments

The improved YOLO-WAD algorithm consisted of four core modules—C2f-WTConv, ASF, C2f-EMA, and DyHead—with each module’s contribution to the YOLO-WAD algorithm’s overall performance being verified through experimental studies, and the experimental results are shown in Table 2.

After the introduction of C2f-WTConv in the backbone part, the models for precision and recall improved by 1.1% and 1.26%, respectively, and mAP@0.5 improved by 1.2%, thus improving the hot-spot detection accuracy by 3.4 %. This indicated that C2f-WTConv could effectively retain more information during the convolution process and enhance the feature extraction ability of the backbone network, thus capturing more defect information features.

After the introduction of ASF in the neck part, the models for precision and recall improved by 1.5% and 2.06%, respectively, and mAP@0.5 improved by 1.5%, thus improving the hot-spot detection accuracy by 4.9 percent. This indicated that the ASF structure effectively fused the different levels of output features extracted from the backbone network and improved the overall feature representation capability of the model.

After the introduction of C2f-EMA in the neck part, the models for precision and recall improved by 1.2% and 3.16%, respectively, and mAP@0.5 improved by 1.8%, thus improving the hot-spot detection accuracy by 5.3 percent. This indicated that the EMA attention mechanism significantly improved the model’s ability to identify and locate the defective parts and strengthened the capture of critical defective features.

After the introduction of DyHead detection in the head section, the models for precision and recall improved by 1.7% and 2.36%, respectively, and mAP@0.5 improved by 1.9%, thus improving the hot-spot detection accuracy by 5 percent. This indicated that DyHead enhanced the model’s ability to classify and localize objects, thus improving the detection accuracy.

Model accuracy was improved in both two-by-two combinations between modules and experiments where the three models were combined together. However, multiple experiments showed that the combination of four modules maximized model performance, with the models for precision and recall improving by 2.7% and 4.56%, respectively, and mAP@0.5 improving by 4.1%, thus improving the hot-spot detection accuracy by 9.5 percent.

These ablation experiments fully demonstrated the significant contribution of each module to the overall performance improvement of the YOLO-WAD algorithm and also verified the rationality and effectiveness of the improvement strategy. The well-designed C2f-WTConv, ASF, C2f-EMA, and DyHead modules enabled YOLO-WAD to capture the target samples more accurately and significantly improved the detection performance, especially in the detection of small-target defects, fully meeting the requirements of industrial production.

## 4. Discussion

Photovoltaic (PV) modules often face a variety of potential faults and defects in actual operation, including small-target defects such as hot spots, which not only lead to energy loss and system efficiency degradation but may also lead to system failure in extreme cases. To address the difficulty of detecting small-target defects in PV modules, this paper proposed an improved YOLO-WAD algorithm based on YOLOv10n. Based on the original YOLOv10n model, the C2f-WTConv, ASF, C2f-EMA, and DyHead modules were elaborately designed, allowing the model to show significantly improved detection precision, recall, and accuracy.

In this paper, the performance of the improved YOLO-WAD algorithm was evaluated against the original YOLOv10n and current mainstream RT-DETR, YOLOv9s, YOLOv8n, and YOLOv11 through comparative experiments. The results showed that YOLO-WAD outperformed all the comparison models in small-target defect detection accuracy, which strongly validated the effectiveness of the algorithm. Meanwhile, the performance of single-module, two–two combinations, and three-module combinations was investigated through ablation experiments, which further proved the robustness and stability of the YOLO-WAD algorithm and showed that the best detection effect was achieved when the four modules worked together.

In other application scenarios, such as in another project by our group on screen defect detection, where the defect categories increase alongside the complexity, defects are often presented as one, several, or many stains or a line, with an accuracy of only about 75%. When detecting defects on these kinds of mobile phone screens, the model cannot clearly identify each and every kind of defect, which may cause false detection. In the future, we will continue to research and optimize our model to overcome this limitation.

## 5. Conclusions

Our research direction is accuracy improvement for small-target defect detection in PV modules. In this study, we compared existing mainstream models, such as RT-DETR-l, RT-DETR-x, RT-DETR-Resnet50, RT-DETR-Resnet101, YOLOv9s, YOLOv8n, and YOLOv11, with our improved YOLO-WAD algorithm, explaining the rationale and reasons for selecting these algorithms. In the “Experimental Results” Section, the detection accuracy of each algorithm was analyzed, and the specific reasons for high or low accuracies were analyzed in depth. In the “Ablation Experiments” Section, we discussed some effects on small-target defects (hot spots) by assessing each module with improved detection, as well as all of them at once. Overall, the results of these experiments showed that the YOLO-WAD algorithm proposed in this paper performed well in the detection of small-target defects in PV modules, with significantly improved accuracy, and could effectively avoid false detections, effectively meeting the needs of industrial production. As such, our algorithm provides new solutions and ideas for relevant fields. In the future, our research will be committed to further optimizing model performance and promoting the continuous development of PV module defect detection technology.

## Figures and Tables

**Table 1:** Performance comparison of different network models.

Algorithm	Precision	Recall	mAP@0.5	Fracture	Hot Spot	Plant	Battery String
YOLOv8n	91.38	84.96	91.57	99.2	75.38	92.2	99.5
YOLOv9s	85.38	89.44	92.7	99.4	78.1	93.7	99.5
YOLOv10n	90.7	88.14	91.5	99.3	76.8	90.5	99.5
YOLOv11	89.89	88.41	91.6	99.3	75.2	92.4	99.5
RT-DETR-l	76.6	76.7	81.4	97.5	64.6	86.7	76.7
RT-DETR-x	79	76.5	81.4	98.5	61.9	86.7	78.4
RT-DETR-Resnet50	82.4	82.5	86	99.2	68.5	88	88.4
RT-DETR-Resnet101	86.5	81	87.5	98.8	71	87.6	92.7
YOLO-WAD (ours)	93.4	92.7	95.6	99.7	86.3	97.5	99.5

**Table 2:** Effectiveness of different modules in detection.

C2f-WTConv	ASF	C2f-EMA	DyHead	Precision (%)	Recall (%)	mAP@0.5 (%)	Hot Spot (%)
				90.7	88.14	91.5	76.8
✓				91.8	89.4	92.7	80.2
	✓			92.2	90.2	93.0	81.7
		✓		91.9	91.3	93.3	82.1
			✓	92.4	90.5	93.4	81.8
	✓	✓		92.8	91.6	94.3	82.6
	✓	✓	✓	93.1	92.3	95.2	84.7
✓	✓	✓	✓	93.4	92.7	95.6	86.3

## Data Availability

The data in this article involves the confidentiality agreement of the relevant cooperation unit, and is not for public use.

## Abbreviations

The following abbreviations have been used in this paper:

Abbreviation	Full name
C2f	CSP bottleneck with two convolutions
WTConv	Wavelet transform convolution
ASF	Attentional scale sequence fusion
EMA	Efficient multi-scale attention mechanism
DyHead	Dynamic head
TFE	Temporal feature extraction
SSFF	Spatial-scale feature fusion
BN	Batch normalization
PV	Photovoltaic
SiLU	Sigmoid-weighted linear unit
C2f-EMA	CSP bottleneck with two convolutions–efficient multi-scale attention
C2f-WTConv	CSP bottleneck with two convolutions–wavelet transform convolution

## References

[1] Zhao S., Chen H., Wang C., Zhang Z. (2023). SSN: Shift suppression network for endogenous shift of photovoltaic defect detection. IEEE Trans. Ind. Inform..

[2] Tsai D.M., Wu S.C., Chiu W.Y. (2012). Defect detection in solar modules using ICA basis images. IEEE Trans. Ind. Inform..

[3] Dhimish M., d’Alessandro V., Daliento S. (2021). Investigating the impact of cracks on solar cells performance: Analysis based on nonuniform and uniform crack distributions. IEEE Trans. Ind. Inform..

[4] Lu C., Zong Y., Zheng W., Li Y., Tang C., Schuller B.W. (2022). Domain invariant feature learning for speaker-independent speech emotion recognition. IEEE/ACM Trans. Audio Speech Lang. Process..

[5] Tsai D.M., Molina D.E.R. (2019). Morphology-based defect detection in machined surfaces with circular tool-mark patterns. Measurement.

[6] Peli T., Malah D. (1982). A study of edge detection algorithms. Comput. Graph. Image Process..

[7] Hu H., Gu J., Zhang Z., Dai J., Wei Y. Relation networks for object detection. Proceedings of the IEEE Conference on Computer Vision and Pattern Recognition, Salt Lake City.

[8] Li Y., Chen Y., Wang N., Zhang Z. Scale-aware trident networks for object detection. Proceedings of the IEEE/CVF International Conference on Computer Vision.

[9] Lin T.Y., Dollár P., Girshick R., He K., Hariharan B., Belongie S. Feature pyramid networks for object detection. Proceedings of the IEEE Conference on Computer Vision and Pattern Recognition.

[10] Liu S., Huang D., Wang Y. (2019). Learning spatial fusion for single-shot object detection. arXiv.

[11] Liu S., Qi L., Qin H., Shi J., Jia J. Path aggregation network for instance segmentation. Proceedings of the IEEE Conference on Computer Vision and Pattern Recognition.

[12] Finder S.E., Amoyal R., Treister E., Freifeld O. (2025). Wavelet convolutions for large receptive fields. Proceedings of the European Conference on Computer Vision.

[13] Kang M., Ting C.M., Ting F.F., Phan R.C.W. (2024). ASF-YOLO: A novel YOLO model with attentional scale sequence fusion for cell instance segmentation. Image Vis. Comput..

[14] Dai X., Chen Y., Xiao B., Chen D., Liu M., Yuan L., Zhang L. Dynamic head: Unifying object detection heads with attentions. Proceedings of the IEEE/CVF Conference on Computer Vision and Pattern Recognition.

[15] Wang H., Yang H., Chen H., Wang J., Zhou X., Xu Y. (2024). A Remote Sensing Image Target Detection Algorithm Based on Improved YOLOv8. Appl. Sci..

[16] Ouyang D., He S., Zhang G., Luo M., Guo H., Zhan J., Huang Z. (2023). Efficient multi-scale attention module with cross-spatial learning. Proceedings of the ICASSP 2023–2023 IEEE International Conference on Acoustics, Speech and Signal Processing (ICASSP).

[17] Gannett P.M., Ye J., Ding M., Powell J., Zhang Y., Darian E., Daft J., Shi X. (2000). Activation of AP-1 through the MAP kinase pathway: A potential mechanism of the carcinogenic effect of arenediazonium ions. Chem. Res. Toxicol..

[18] Zhao Y., Lv W., Xu S., Wei J., Wang G., Dang Q., Liu Y., Chen J. Detrs beat yolos on real-time object detection. Proceedings of the IEEE/CVF Conference on Computer Vision and Pattern Recognition.

[19] Wang C.Y., Yeh I.H., Mark Liao H.Y. (2025). Yolov9: Learning what you want to learn using programmable gradient information. Proceedings of the European Conference on Computer Vision.

[20] Varghese R., Sambath M. (2024). YOLOv8: A Novel Object Detection Algorithm with Enhanced Performance and Robustness. Proceedings of the 2024 International Conference on Advances in Data Engineering and Intelligent Computing Systems (ADICS).

[21] Khanam R., Hussain M. (2024). Yolov11: An overview of the key architectural enhancements. arXiv.

